# Nocardia cyriacigeorgica Brain and Lung Abscesses in 77-Year-Old Man With Diabetes

**DOI:** 10.7759/cureus.19373

**Published:** 2021-11-08

**Authors:** Weston D Browne, Robert E Lieberson, Mohammad J Kabbesh

**Affiliations:** 1 Neuroscience and Biology, Santa Clara University, Santa Clara, USA; 2 Clinical Research, Dignity Health, Sacramento, USA; 3 Research Associate I in Neurology, University of California San Francisco, San Francisco, USA; 4 Department of Neurosurgery, Mercy General Hospital, Sacramento, USA; 5 Department of Infectious Diseases, Mercy General Hospital, Sacramento, USA

**Keywords:** nocardia cyriacigeorgica, brain abscess, infectious disease, actinomyces, aerobic bacteria, lung abscess, nocardia, diabetes, tobacco, gram-positive bacteria

## Abstract

*Nocardia* species are difficult to identify, saprophytic, aerobic actinomycetes that are associated with high mortality rates and primarily affect immunocompromised hosts. Recently, the number ofdiagnoses of *Nocardia cyriacigeorgica *has grown in the United States, yet complicated clinical diagnosis and costly identification methods presume an underestimation of its presence in patients. We describe a case of brain abscess secondary to a pulmonary infection in an elderly, diabetic, Afghani man with an extensive history of chewing tobacco use.

## Introduction

*Nocardia* are non-motile, Gram-positive, partially acid-fast bacilli, most commonly responsible for lung, brain, and skin infections in immunocompromised hosts including those with acquired immunodeficiency syndrome (AIDS), cancer, lupus, and diabetes. Infections in immunocompetent hosts have been well reported [1-2]. First isolated in 2001 by Yassin et al., *Nocardia cyriacigeorgica* is difficult to identify and differentiate from nontuberculous mycobacteria using conventional methods making clinical diagnosis elusive [1-3]. Increased susceptibility to infection by this bacterium has been proven in diabetics and patients who use immunosuppressive drugs [4]. Increasing incidence rates in the United States have been attributed to travel and immigration [2]. Reports have also demonstrated the presence of Gram-positive bacteria in dried foliage, such as marijuana flower, and exposure by inhalation has been linked to infection [5]. Gene sequencing remains one of the only conclusive techniques to identify characteristic genes such as 16S rRNA in *N. cyriacigeorgica* [1,6].

## Case presentation

A 77-year-old Afghani male presented with worsening cough and headache of four months’ duration, associated with fever, blurred vision, dysphonia, instability of gait, and impaired consciousness. An immigration screening exam shortly before symptoms began and prior to arrival in the United States was reportedly negative. He provided a history of a presumed cerebrovascular accident with right arm weakness three years earlier in Afghanistan but denied imaging performed to confirm the diagnosis. His past medical history was otherwise notable for untreated hypertension and diabetes. He endorsed to chewing tobacco daily. He denied history of travel beyond southwest Afghanistan and Northern California, as well as outdoor work, animal contact, sinus complaints, and dental disease.

On examination, the patient was afebrile with stable vital signs. He was awake and oriented in his native language, Dari, but had difficulty finding words. Mentation was slow and he was unable to follow multistep commands. The patient presented with a right facial droop, an incomplete right hemisensory loss, and 3-4/5 weakness on the right extremity with brisk reflexes.

**Figure 1:**
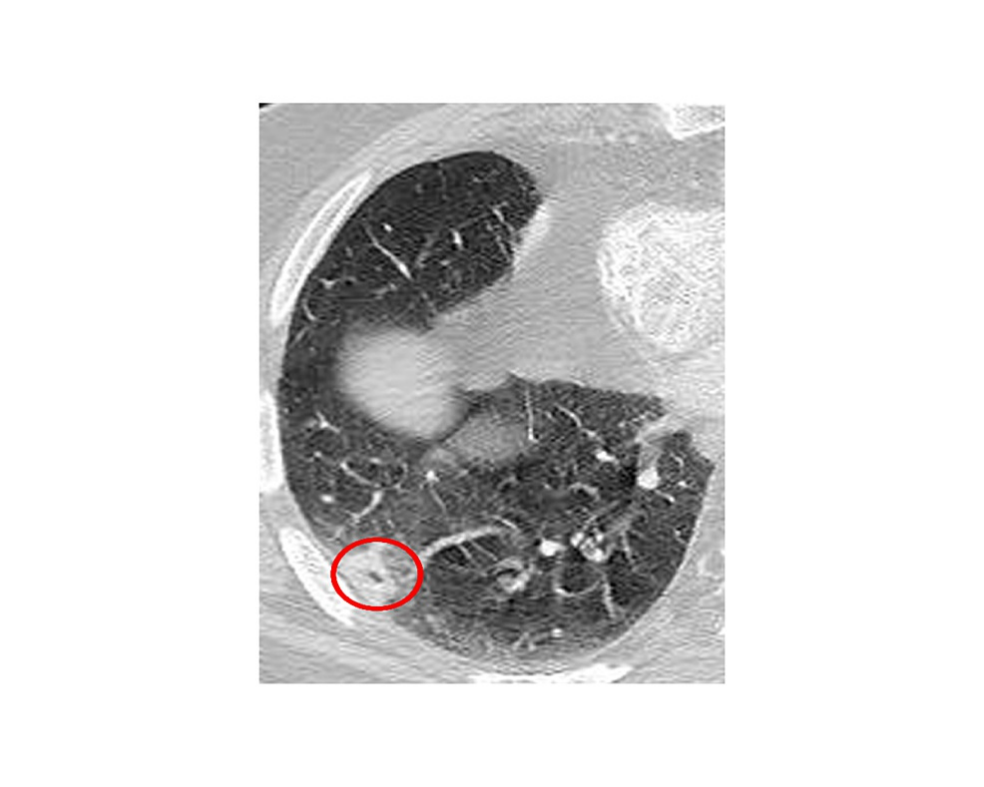
Inferior chest X-ray. CT image of a lung demonstrating right-sided anterior cavitary lung lesion encircled in red.

**Figure 2:**
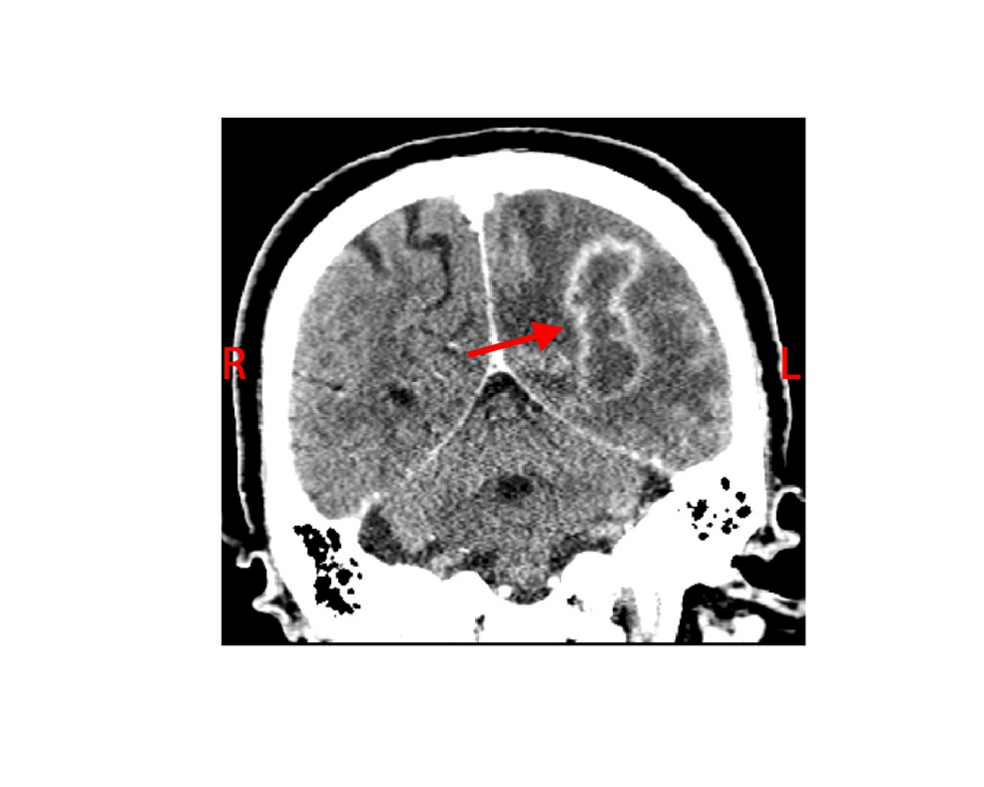
Coronal brain CT demonstrated (red arrow) left parieto-occipital lesion.

**Figure 3:**
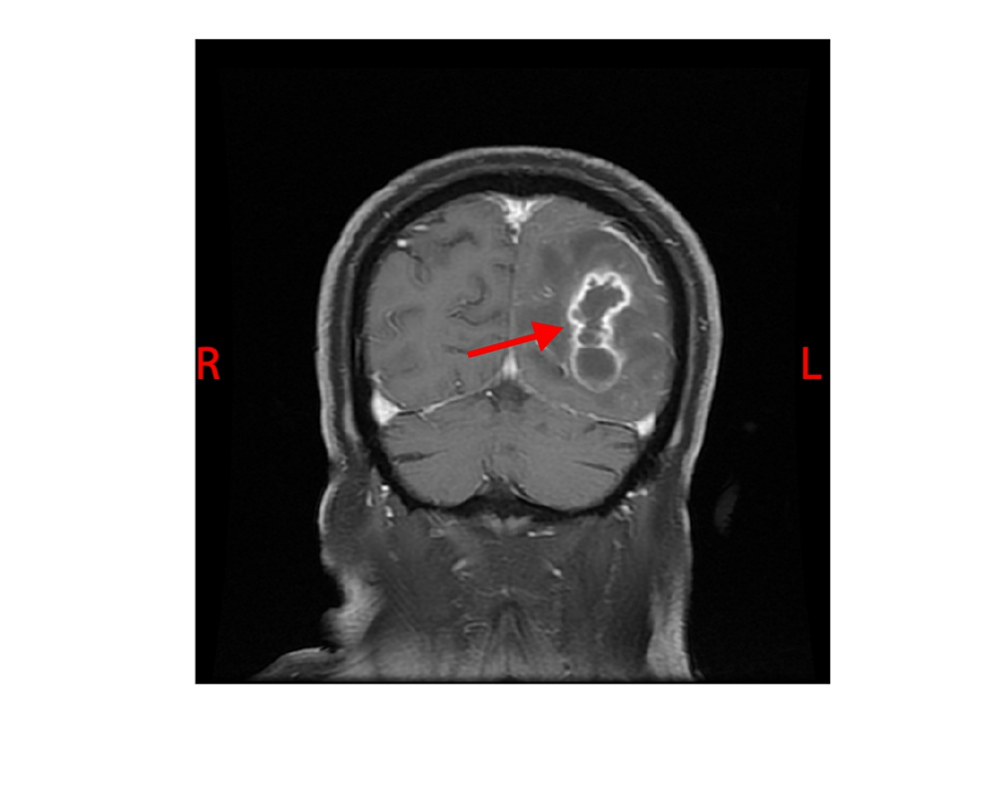
Coronal brain MRI with contrast demonstrated (red arrow) left parieto-occipital lesion.

The white blood cell count and comprehensive metabolic panel were unremarkable. His hemoglobin A1c was 5.6 (normal range 4-5.6). Human immunodeficiency virus testing was negative. A chest CT showed a right, pleural-based, cavitary lung lesion (Figure 1). CT head (Figure 2) and MRI (Figure 3) showed a 2.9-cm, ring-enhancing, left parieto-occipital lesion with significant edema and mass effect. A stereotactic needle aspiration found gross pus. Empiric antibiotics were started, and the patient’s neurological examination rapidly improved on intravenous (IV) ceftriaxone, oral trimethoprim-sulfamethoxazole, and a tapering course of dexamethasone.

**Figure 4:**
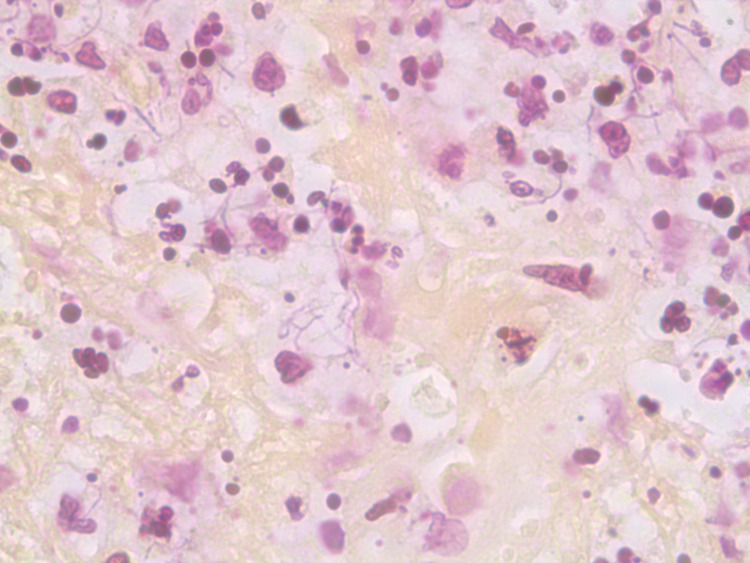
Gram stain (10x40) depicting the presence of Gram-positive bacteria (purple).

**Figure 5:**
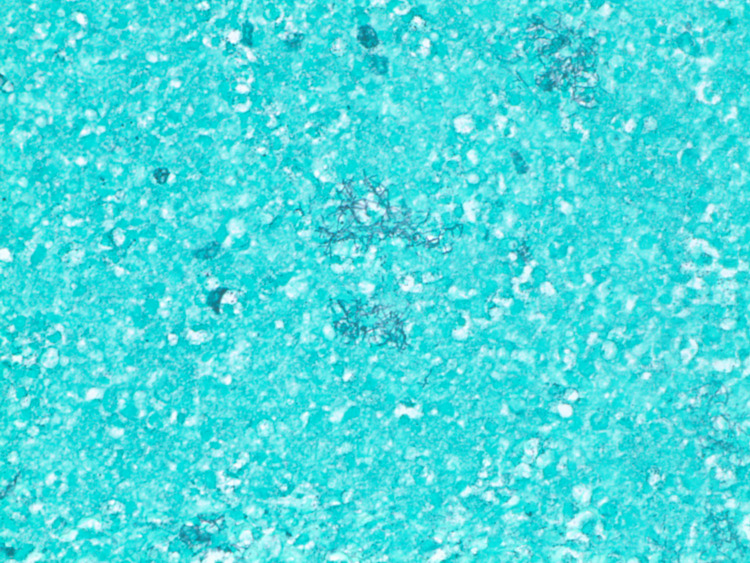
Grocott’s methenamine silver (GMS) (10x100) stain depicting filamentous bacteria (dark blue).

**Figure 6:**
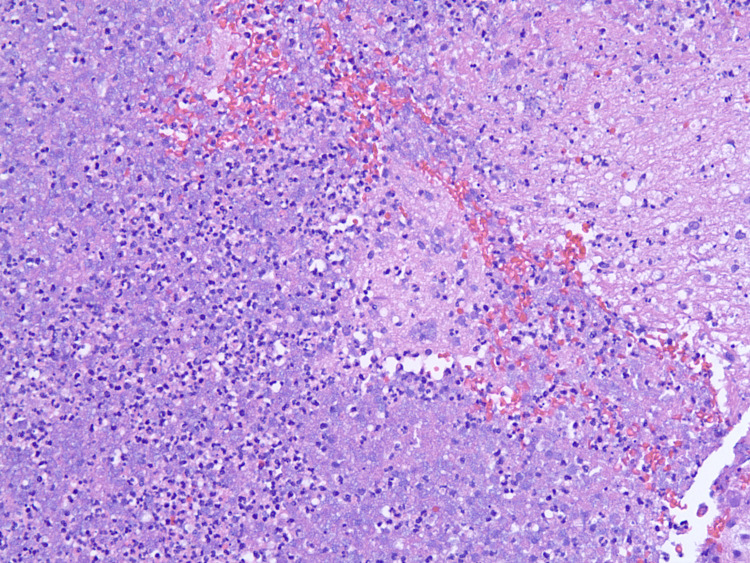
Hematoxylin and eosin (H&E) (10x20) stain depicting purulence found in surgery.

**Figure 7:**
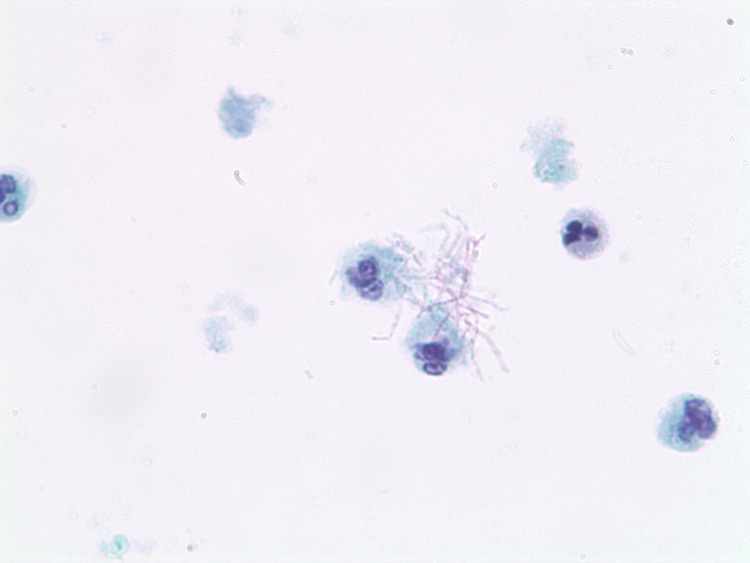
ThinPrep-PaP (10x100) depicting N. cyriacigeorgica with pink filamentous structures and purple nuclei.

Sensitivity reviewal (Table 1) confirmed the beneficence of the antibiotics and a treatment course was planned. The laboratory performed cytologic preparation (Figure 7) in addition to Gram (Figure 4), Grocott’s methenamine silver (GMS) (Figure 5), and hematoxylin & eosin (HE) stains (Figure 6) on the sample, and the findings showed filamentous properties and moderate, branching, Gram-positive rods. Methicillin-resistant *Staphylococcus aureus* (MRSA), ova and parasites, and fungal elements were not identified. Cultures eventually grew *N. cyriacigeorgica* and were determined by sequence analysis to possess the 16S rRNA gene.

**Table 1:** Sensitivities of Nocardia cyriacigeorgica to antibiotics

Antibiotic	ug/mL	Interpretation
Trimethoprim/Sulfamethoxazole	≤0.28/4.8	Susceptible
Ciprofloxacin	≥8	Resistant
Moxifloxacin	2	Intermediate
Amikacin	≤1	Susceptible
Doxycycline	2	Intermediate
Clarithromycin	≥32	Resistant
Linezolid	2	Susceptible
Imipenem	4	Susceptible
Amoxicillin/Clavulanate	32/16	Resistant
Ceftriaxone	8	Susceptible
Minocycline	2	Intermediate
Tobramycin	≤1	Susceptible

## Discussion

The most frequently involved *spp. of Nocardia* in human infections are members of the *Nocardia asteroides* complex, which have been subclassified into six different drug susceptibility types. Assessed by Wallace et al. via broth microdilution, members of *N. asteroides* possess characteristic resistance to penicillin, ciprofloxacin, and clarithromycin, with susceptibility to broad-spectrum cephalosporins, amikacin, imipenem, and linezolid. Wallace et al. determined that 35% of all clinical isolates had this antimicrobial susceptibility pattern and identified it as drug pattern type VI [7]. Yassin et al. isolated *Nocardia* with drug pattern type VI by using 16S rRNA [3]. 65-kDa heat shock protein polymerase chain reaction and sequence analysis determined these to be a new species, *N. cyriacigeorgica*. Since then, reviews of clinical *Nocardia* isolates utilizing molecular methods have identified a significant proportion of *N. asteroides* complex isolates to be *N. cyriacigeorgica* [8].

*Nocardia* can be found worldwide as saprophytic components in fresh- and saltwater, soil, dust, and decaying vegetation [9-10]. Inhalation remains the most commonly attributed route of infection and seems to be the major cause of pulmonary nocardiosis [2]. Saubolle and Sussland report that *Nocardia* infections in the United States are more prevalent in arid, warm climates [10-11]. Additionally, Larsson et al. reported the presence of Gram-positive bacteria and multiple *Bacillus spp.* in dried tobacco leaves from the United States in 2008 and soil has been found to contain *N. cyriacigeorgica*, indicating that this species of bacteria could be transmitted by pulmonary exposure to tobacco containing the organism. The warm-arid environments, in which our Afghani patient’s tobacco was grown, are likely to have a greater prevalence of *N. Cyriacigeorgica*. Recently Chavez et al. reported the first case of *N. cyriacigeorgica* in a 58-year-old male working in Afghanistan [5,11-12].

Reports have additionally linked patients with chronic granulomatous disease (CGD) to infection by *N. cyriacigeorgica* [13]. In 2008, Latif et al. reported an *N. cyriacigeorgica* empyema in a 45-year-old male with dual granulomatous lung disease [13]. CGD is characterized by defective phagocyte nicotinamide adenine dinucleotide phosphate (NADPH) oxidase function resulting in susceptibility to severe and recurrent infections as well as granuloma formation; it is the most common symptomatic phagocyte defect. CGD is induced by mutations in genes encoding protein subunits of the NADPH oxidase complex from an inherited X-linked recessive or autosomal recessive pattern. Nitroblue-tetrazolium dye reduction test and dihydro-rhodamine (DHR) assay by flow cytometry are the screening tests for this disorder and are commonly run on patients with nocardial infections to assess comorbidity as well as immunocompetence [14-16].

DHR by flow cytometry is performed by stimulating white blood cells and measuring DHR oxidation to rhodamine by the respiratory burst of the cell. To assess our patient for CGD, neutrophil samples were sent to Associated Regional and University Pathologists (ARUP) Laboratories and DHR by flow cytometry was performed yielding results of both an intermediate (57.7%) and a normal (41.9%) population of granulocyte DHR fluorescence, supporting a diagnosis of CGD.

## Conclusions

*N. cyriacigeorgica* is an emerging pathogen in the United States. Previously reported as a complication of diabetes and following the use of immunosuppressive medications, it has not been reported with tobacco use. *N. cyriacigeorgica*, a common soil contaminant in Afghanistan, would be an expected contaminant in that region and is the likely source of the organism.
